# Modulation of chemokine secretion and oxidative stress *in vitro* in thyroid cells after exposure to thiocyanate, nitrate, or perchlorate

**DOI:** 10.3389/fendo.2026.1787679

**Published:** 2026-05-22

**Authors:** Alessia Greco, Francesca Coperchini, Elena Franchi, Marco Denegri, Laura Croce, Marsida Teliti, Vincenzo Marotta, Flavia Magri, Giuseppina De Marco, Eleonora Ferrarini, Luisa Pignata, Patrizia Agretti, Massimo Tonacchera, Mario Rotondi

**Affiliations:** 1 Department of Internal Medicine and Therapeutics, University of Pavia, Pavia, Italy; 2 Ph.D in Internal Medicine and Therapeutics, University of Pavia, Pavia, Italy; 3 Unit of Endocrinology and Metabolism, Laboratory for Endocrine Disruptors, Istituti Clinici Scientifici Maugeri Istituto di Ricovero e Cura a Carattere Scientifico (IRCCS), Pavia, Italy; 4 Department of Medicine, Surgery and Dentistry, “Scuola Medica Salernitana”, University of Salerno, Baronissi, Italy; 5 Department of Clinical and Experimental Medicine, Endocrine Unit, University of Pisa, Pisa, Italy; 6 Laboratory of Chemistry and Endocrinology, University Hospital of Pisa, Pisa, Italy

**Keywords:** CXCL10, CXCL8, nitrate, perchlorate, ROS, thiocyanate, thyroid

## Abstract

**Background:**

Perchlorate, nitrate, and thiocyanate are thyroid disruptors. Emerging evidence suggests they may exert further effects independent of iodide uptake inhibition, influencing oxidative stress and thyroid tumour development. This study aimed to assess their effects on cell viability, proliferation, reactive oxygen species (ROS) production, and chemokine expression in normal human thyroid cells and thyroid cancer cell lines.

**Methods:**

Thyroid cells, both normal (NHT, in primary cultures) and cancer (TPC-1 and 8505C), were exposed to increasing concentrations of perchlorate, nitrate, or thiocyanate for 24 hours. Cell viability and proliferation were assessed in each cell type. Intracellular ROS production was measured using H_2_DCFDA. CXCL8 and CXCL10 expression was evaluated at both mRNA (RT-PCR) and protein levels (ELISA in cell supernatants).

**Results:**

None of the three anions significantly affected viability in NHT or 8505C cells, whereas high-dose thiocyanate reduced viability in TPC-1 cells. Proliferative responses were modest and cell-type specific, occurring mainly at the highest concentrations tested. Perchlorate induced a biphasic increase in ROS production in NHT, while thiocyanate slightly reduced ROS levels in TPC-1 cells; no consistent effects were observed in 8505C cells. Thiocyanate consistently increased CXCL8 secretion in NHT and TPC-1 cells, with a concentration-dependent but non-monotonic pattern, whereas nitrate and perchlorate had minimal effects. CXCL10 protein was undetectable in all conditions, despite significant and cell-specific modulation of CXCL10 mRNA expression.

**Conclusions:**

Beyond their role as NIS inhibitors, perchlorate, nitrate and thiocyanate modulate oxidative stress and chemokine secretion in human thyroid cells. Thiocyanate promotes a pro-inflammatory phenotype, potentially favouring a tumour-promoting thyroid microenvironment.

## Introduction

Perchlorate, nitrate, and thiocyanate are well-known thyroid disruptors that interfere with the transport of iodide into the thyroid gland by inhibiting the sodium-iodide symporter (NIS). This, in turn affects the synthesis of thyroid hormones (1). Perchlorate is a natural substance (i.e., atmospheric oxidation of chloride, deposits in arid soils) but also derives from industrial sources (i.e., rocket propellant, explosives), mainly found in drinking water and various commercial products (2). Nitrate occurs naturally in the nitrogen cycle and through lightning, and is also produced by fertilizers and wastewater, thus can be found in air, soil, water, and food (3, 4). Thiocyanate is produced in the body from the metabolism of certain cyanogenic plants and it is also released from industrial wastes such as mining and coal gasification (5–7). Exposure to thiocyanate mainly occurs through smoking (8) and dietary sources, such as cruciferous vegetables, almonds, beans, and cassava (9). Recent studies demonstrated that several endocrine disruptors (EDs) once considered “non-carcinogenic” have subsequently shown potential tumour-promoting effects. For instance, bisphenol-A and polybrominated diphenyl ethers have been associated with increased proliferation and invasiveness in breast and thyroid cancer cell models (10), while PFAS exposure was associated with a higher risk of several malignancies, including testicular, kidney, and thyroid cancer (11). Recent epidemiological studies have linked exposure to thiocyanate, nitrates or perchlorate increased risk of cancer including thyroid cancer (12–15). Thyroid tumour microenvironment (TME) and oxidative stress tumorigenesis strongly influence thyroid tumour progression. The TME is composed of a complex network of pro-tumorigenic and anti-tumorigenic factors (including various cells and chemokines), that interact to regulate cell proliferation, angiogenesis, metastasis and immune modulation. An imbalance in these signals, particularly when there is an excess of pro-tumorigenic factors, is associated with more aggressive disease and a worse prognosis (16). CXCL8 is a pro-tumorigenic chemokine with a well-established role in thyroid cancer, where its higher levels are associated with metastatic processes both *in vitro* and *in vivo*. Thyroid tumorigenesis is also influenced by excessive reactive oxygen species (ROS), which, while normally aiding thyroid hormone biosynthesis, can cause DNA damage, genomic instability, and cell de-differentiation, contributing to cancer progression (17). A recent *in vitro* study found that exposure to some thyroid disruptors alters pro-tumorigenic chemokine secretion, including CXCL8, and increases ROS production in human thyroid cells (18).

The aim of the present study was to investigate the effects of perchlorate, nitrate, and thiocyanate on cell viability, proliferation, ROS production, CXCL8 and CXCL10 expression in primary human thyrocytes (NHT) and in two thyroid cancer cell lines: TPC-1 (papillary thyroid carcinoma) and 8505C (undifferentiated/anaplastic thyroid carcinoma).

## Materials and methods

### Primary cultures of normal human thyroid cells

Primary cultures were derived from human thyroid surgical specimens from three patients undergoing thyroidectomy for goitre or solitary nodule. The normal tissue used for the experiments was derived from the healthy contralateral thyroid lobe with approval from the Institutional Review Board of ICS-Maugeri in Pavia, Italy. Written consent has been obtained from each patient or subject after full explanation of the purpose and nature of all procedures used, and experiments followed the Declaration of Helsinki guidelines.

The tissue was delivered to the Pathology Unit immediately after surgical excision without any medium (dry) at room temperature (r.t.); there, a part of the specimen was collected for our experiments. All these procedures usually take 10 minutes, before proceeding with the isolation protocol. Primary cultures were obtained based on the following protocol performed under biological hood at r.t.: i) shredding of tissues with a sterile scalpel, ii) incubation in collagenase (type II, 5 mg/ml, Sigma, Saint Louis, MO, USA) for 4 h at 37 °C (19), iii) filtering of the obtained homogenate with Corning Cell Strainer 70 µm, nylon (Corning, REF 431751), iv) spun at 1000 rpm for 10 minutes at r.t. washed twice with 10 ml of Coon’s F-12 medium (1.34 g of Sodium-hydrogen-carbonate (Applichem CAS 144-55-8), 5.75 g of Nutrient Mixture F-12 Ham (Sigma-Aldrich, NG760), 500 ml of sterile water, then sterile filtered), v) resuspended in Coon’s 6H medium (Coon’s F-12 medium supplemented by Insulin 0.1 mg/ml, Hydrocortisone 1 mg/ml, Apo-Transferrin 0.1 mg/ml, Somatostatin 0.2 mg/ml, Glycine-Histidine-Lysine 0.2 mg/ml, TSH 1 mU/ml and 5% newborn calf serum) (20, 21). All the cells obtained were seeded in a 60 × 15 mm cell culture dish (SPL Life Sciences, 20060, crystal grade polystyrene, tissue culture treated and sterilized). After 24 hours cells were detached by 0.5% trypsin (Corning, REF 25-053-CI) at 37 °C for 3 minutes in order to be used for experiments. Cells were then counted by a Cell Counter (NanoEntek EVE-Plus, EVE-MC2). After count, only cell cultures with a viability above 87% were used for subsequent experiments. Cells were seeded (always starting from the number of viable cells, not the total counting number) at the specific density necessary for each assay (below better described).

### Cultures of thyroid tumour cell lines

8505C (CVCL_1054, BRAF V600E mutation), and TPC-1 (CVCL_6298, RET/PTC mutation) are human thyroid cancer cell lines previously authenticated by DNA analysis. TPC-1 cell line was purchased from Cytion (Cat No. 305054), while, 8505C cell line was purchased from AcceGen (Cat No. ABC-TC0020). Medium required for cultures were Dulbecco’s modified Eagle’s medium (DMEM, Sigma, Saint Louis, MO, USA) and RPMI (Sigma, Saint Louis, MO, USA) for TPC-1 and 8505C, respectively. Both media were supplemented with 10% foetal bovine serum (Sigma, Saint Louis, MO, USA), 2 mM l-glutamine, and 100 U/ml penicillin/streptomycin (Sigma, Saint Louis, MO, USA) (22). All the experiments were performed with cell lines at passages between 10 and 25 after thawing. Mycoplasma test is routinely performed every two months.

### WST-1 for the viability assay

2 x 10^4^ cells/well were seeded in a 96 well flat as previously reported (22) and treated with increasing concentrations of thiocyanate (KSCN; CAS No. 333-20-0, Cat No. P3011, Sigma-Aldrich), nitrate (KNO_3_; CAS No. 7757-79-1, Cat No. PHR2203, Sigma-Aldrich), or perchlorate (NaClO_4_; CAS No. 7601-89-0, Cat No. 410241, Sigma-Aldrich). Thiocyanate, nitrate and perchlorate stocks in sterile water are highly soluble in water and were dissolved in deionized water to prepare stock solutions, which were subsequently diluted to obtain the desired working concentrations (0; 0.01; 0.1; 1; 10; 100 µM). The range of concentrations was chosen according to biomonitoring, human and ambient matrices concentrations (23–30). After 24 hours, cells were incubated with WST-1 at 37 °C for 30 minutes (absorbance read at 450nm, Victor NIVO Multimode Plate Reader, PerkinElmer). All measurements were performed in at least three independent experiments, each in technical triplicate.

### Cell proliferation assay

3000 cells per well were incubated with thiocyanate, nitrate, or perchlorate at increasing concentrations (0; 0.01; 0.1; 1; 10; 100 µM). After 24 hours, cells were fixed with methanol and stained with 0.5% crystal violet dye (C0775; Sigma-Aldrich) as previously described (31). 1% sodium dodecyl sulfate (SDS) (436143; Sigma-Aldrich) was added to induce the release of crystal violet dye (absorbance measured at 570 nm). All measurements were performed in at least three independent experiments, each in technical triplicate.

### Detection of reactive oxygen species production

3 x 10^4^ cells/well were seeded in a 96 well flat and were exposed to increasing concentrations of thiocyanate, nitrate, or perchlorate (0; 0.01; 0.1; 1; 10; 100 µM) for 24 hours. To assess the production of reactive oxygen species (ROS) by NHT, TPC-1 and 8505C, we used the cell-permeant 2’,7’-dichlorodihydrofluorescein diacetate (H_2_DCFDA) (Sigma Aldrich). At the end of the incubation period, H_2_DCFDA was added for 45 minutes, and positive control cultures were treated with H_2_O_2_. After washing the cells with PBS, fluorescence was measured using a microplate reader (excitation 492–495 nm; emission 517–527 nm) (Victor Nivo, PerkinElmer). ROS production was derived from OD measurements, which were normalized to the untreated control, which was set as 100%, and results were expressed as a percentage of ROS relative to the control. All measurements were performed in at least three independent experiments, each in technical triplicate.

### Real-time PCR experiments

Cells were seeded at a density of 1 x 10^5^ in a 6 well and incubated with thiocyanate, nitrate, or perchlorate at the concentrations of 1 and 100 µM for 24 hours. RNA was extracted using the Total RNA Purification Kit (Norgen Biotek, Canada, Cat. 17200) with DNasi I kit (Norgen Biotek, Canada, Product 25720) and 0.5 µg/µl were reverse transcribed with a kit for the synthesis of cDNA (SensiFast, Bioline, London, UK). RT-PCRs were performed using SYBR Green (Sensi-Fast SYBR Green Hi-ROX kit, Bioline, London, UK) on a real-time PCR system (StepOne Plus, Thermofisher). Pre-designed primers targeting *CXCL8* (F: CAGTGCATAAAGACATACTC; R: CTCTTCAAAAACTTCTCCAC) NM_000584: FOR cDNA position 200-212; REV cDNA position 374-353 (Tm = 64 °C); and *CXCL10* (F: TGCCATTCTGATTTGCTGCC; R: TGATGGCCTTCGATTCTGGA) NM_001565.4: FOR position 79-99, REV position 315-296 (Tm = 60 °C); were all HPLC purified and purchased from Biomers.net GMBH (Soflinger, Germany). *GAPDH* (F: AAATCCCATCACCATCTTCC; R: GGTTCACACCCATGACGAAC) NM_002046.7: FOR cDNA position 289-308; REV cDNA position 485–466 was selected as the reference gene. Gene expression levels were quantified using the comparative Ct (ΔΔCt) method. Ct values of target genes were first normalized to the housekeeping gene *GAPDH* (ΔCt), and then compared to the ΔCt of control samples to obtain relative expression levels (ΔΔCt). Final results were expressed as fold change (2^-ΔΔCt^) relative to untreated control cells set as 1, as they serve as the reference condition for comparison. All measurements were performed in at least three independent experiments, each in technical triplicate.

### Enzyme linked immunosorbent assay for CXCL8 and CXCL10

3000 cells were incubated in medium serum-free without (basal condition) or with increasing concentrations (0.01; 0.1; 1; 10; 100 µM) of thiocyanate, nitrate, or perchlorate. Experiments were performed in triplicates. In thyroid cultures cell supernatants, CXCL8 and CXCL10 levels were measured by using ELISA kits (R&D Systems, Minneapolis, MN). Samples were assayed in duplicates.

### Cells migration

Trans-well migration chamber system (Merck Millipore, Milan, Italy) was employed for migration assay. 10^5^ cells (NHT, TPC-1 and 8505C) were cultured for 24 hours with fresh medium alone or supplemented with thiocyanate 1 µM (for 8505C) and 100 µM (for NHT and TPC-1). The concentrations were selected based on ELISA results, showing the greatest increase in CXCL8 secretion after exposure to thiocyanate. After the treatment, 30 x 10^3^ cells/well were seeded in the upper chambers of the 24-well plate with polycarbonate inserts having 0.3 cm^2^/well membrane area and 8 μm pore size. In each condition the lower chambers were filled with 500 μl of the corresponding medium. Cells were left to migrate for 16 hours at 37 °C and 5% CO2. At the end of the incubation, cell inserts were washed three times with PBS and migrated cells on the underside of the membrane were fixed with 4% paraformaldehyde for 10 min. Cell nuclei were then stained with Hoechst 33342 (1:2000) (Life Technologies, Monza, Italy). Finally, the membranes were cut out with a scalpel, and mounted onto glass slides. Images were acquired using an Olympus BX51 microscope (Olympus, Deutschland GmbH, Hamburg, Germany). The number of migrated cells was counted analysing 12 random fields of the membranes per condition.

### Statistical analysis

The software SPSS (SPSS, Inc., Evanston, IL) was used for statistical analyses. Results of detection of viability, proliferation, ROS and ELISA assays were entered into separate analyses of variance (one-way ANOVA) for the comparison of Mean group values. *Post hoc* analysis was then performed (*p-value* was assigned according to Bonferroni correction for multiple comparisons). For mRNA analysis, Kruskal-Wallis for independent samples was used (*p*-value was assigned according to Bonferroni correction for multiple comparisons). For migration assay, Wilcoxon for independent samples was used.

## Results

### Effect of thiocyanate, nitrate, or perchlorate on thyroid cells viability

**Figure 1:**
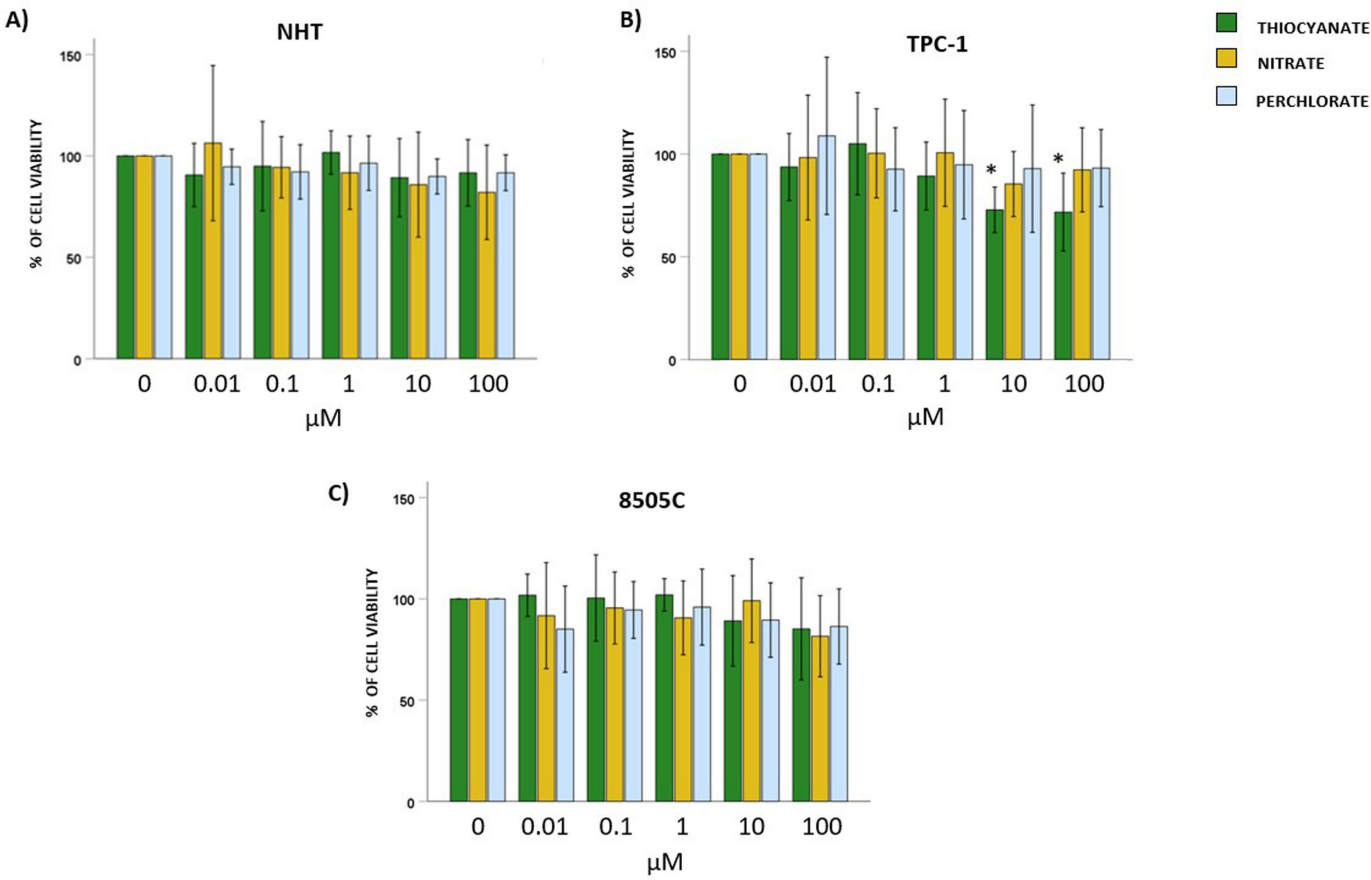
Effect of thiocyanate, nitrate and perchlorate on thyroid cell viability. Results of WST-1 employed to assess changes in viability on NHT, TPC-1 and 8505C cells. Bar graphs: y-axis indicates the % of cell viability; x axis indicate the concentrations of thiocyanate, nitrate and perchlorate (µM). Bars show the effect of thiocyanate (green bars), nitrate (yellow bars) and perchlorate (blue bars). **(A)** shows the % of cell viability after treating NHT cells with increasing concentration of thiocyanate, nitrate or perchlorate. **(B)** shows the % of cell viability after treating TPC-1 cells with increasing concentration of thiocyanate, nitrate or perchlorate. **(C)** shows the % of cell viability after treating 8505C cells with increasing concentration of thiocyanate, nitrate or perchlorate. Bars represent the mean ± SD of normalized values. Control (0 µM) values were set to 100% after normalization; as a result, SD is not shown for control bars. Results were expressed as % of cell viability relative to control. Significant changes between treated samples *vs.* controls were indicated by *.

Normal Human thyroid cells (NHT), and TC cell lines TPC-1 and 8505C, were exposed to increasing concentrations (0; 0.01; 0.1; 1; 10; 100 µM) of thiocyanate, nitrate, or perchlorate for 24 hours. The treatment of NHT with thiocyanate, nitrate or perchlorate did not modify cell viability at all concentrations tested (ANOVAs: thiocyanate F = 1.617, *p*=NS; nitrate F = 2.229, *p*=NS; perchlorate F = 2.090, *p*=NS) (**Figure 1A**).

Moving to TC cell lines, the exposure of TPC-1 to thiocyanate at the highest concentration of 10 and 100 µM reduced cell viability after 24 hours (ANOVA: thiocyanate F = 8.422, *p* < 0.05, *post hoc* 10 µM and 100 µM *vs.* 0 µM, *p* < 0.05). No significant changes in TPC-1 viability were observed after treatment with either nitrate (ANOVA: nitrate F = 0.984, *p*=NS) or perchlorate (ANOVA: perchlorate F = 0.760, *p*=NS) (**Figure 1B**). The exposure of 8505C to these anions showed no significant changes in cell viability (ANOVAs: thiocyanate F = 1.462, *p*=NS; nitrate F = 1.026, *p*=NS; perchlorate F = 0.978, *p*=NS) (**Figure 1C**).

### Effect of thiocyanate, nitrate, or perchlorate on thyroid cells proliferation

**Figure 2:**
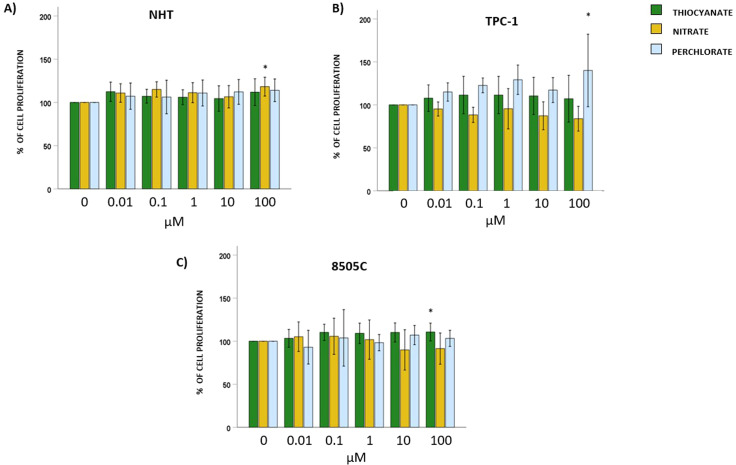
Effect of thiocyanate, nitrate and perchlorate on thyroid cells proliferation. Results of crystal violet assay employed to assess changes in the proliferation of thyroid cells. Bar graphs: y-axis indicates the % of cell proliferation; x-axis indicate the concentrations of thiocyanate, nitrate or perchlorate (µM). Bars show the effect of thiocyanate (green bars), nitrate (yellow bars) and perchlorate (blue bars) on cell proliferation. **(A)** shows the % of cell proliferation after treating NHT cells with increasing concentrations of thiocyanate, nitrate or perchlorate. **(B)** shows the % of cell proliferation after treating TPC-1 cells with increasing concentration of thiocyanate, nitrate or perchlorate. **(C)** shows the % of cell proliferation after treating 8505C cells with increasing concentration of thiocyanate, nitrate or perchlorate. Bars represent the mean ± SD of normalized values. Control (0 µM) values were set to 100% after normalization; as a result, SD is not shown for control bars. Results were expressed as % of cell proliferation relative to control. Significant changes between treated samples *vs.* controls were indicated by *.

NHT, and TC cell lines TPC-1 and 8505C were exposed to increasing concentrations (0; 0.01; 0.1; 1; 10; 100 µM) of thiocyanate, nitrate, or perchlorate for 24 hours. The exposure of NHT to thiocyanate, or perchlorate did not modify cell proliferation at all concentrations tested (ANOVAs: thiocyanate F = 2.175, *p*=NS; perchlorate F = 1.534, *p*=NS), while exposure to nitrate led to a significant increase in NHT proliferation only at the highest concentration of 100 µM (ANOVA: nitrate F = 4.841, *p* < 0.05, *post hoc* Bonferroni 100 µM *vs*. 0 µM, *p* < 0.05) (**Figure 2A**).

Moving to TC cell lines, starting from TPC-1, treatment with thiocyanate and nitrate did not change cell proliferation (ANOVAs: thiocyanate F = 0.527, *p*=NS; nitrate F = 2.201, *p*=NS). On the other hand, the exposure to perchlorate significantly increase cell proliferation only at the highest concentration of 100 µM (ANOVA: perchlorate F = 3.301, *p* < 0.05; *post hoc* Bonferroni 100 µM *vs.* 0 µM, *p* < 0.05) (**Figure 2B**). In 8505C, exposure to nitrate and perchlorate did not induce significant changes in cell proliferation (ANOVAs: nitrate F = 2.051, *p*=NS; perchlorate F = 0.980, *p*=NS), whereas exposure to thiocyanate led to a significant increase in cell proliferation only at the highest concentration of 100 µM (ANOVA: thiocyanate F = 3.226, *p* < 0.05; *post hoc* Bonferroni 100 µM *vs.* 0 µM, *p* < 0.05) (**Figure 2C****).**

### Effect of thiocyanate, nitrate, or perchlorate on oxidative stress production

**Figure 3:**
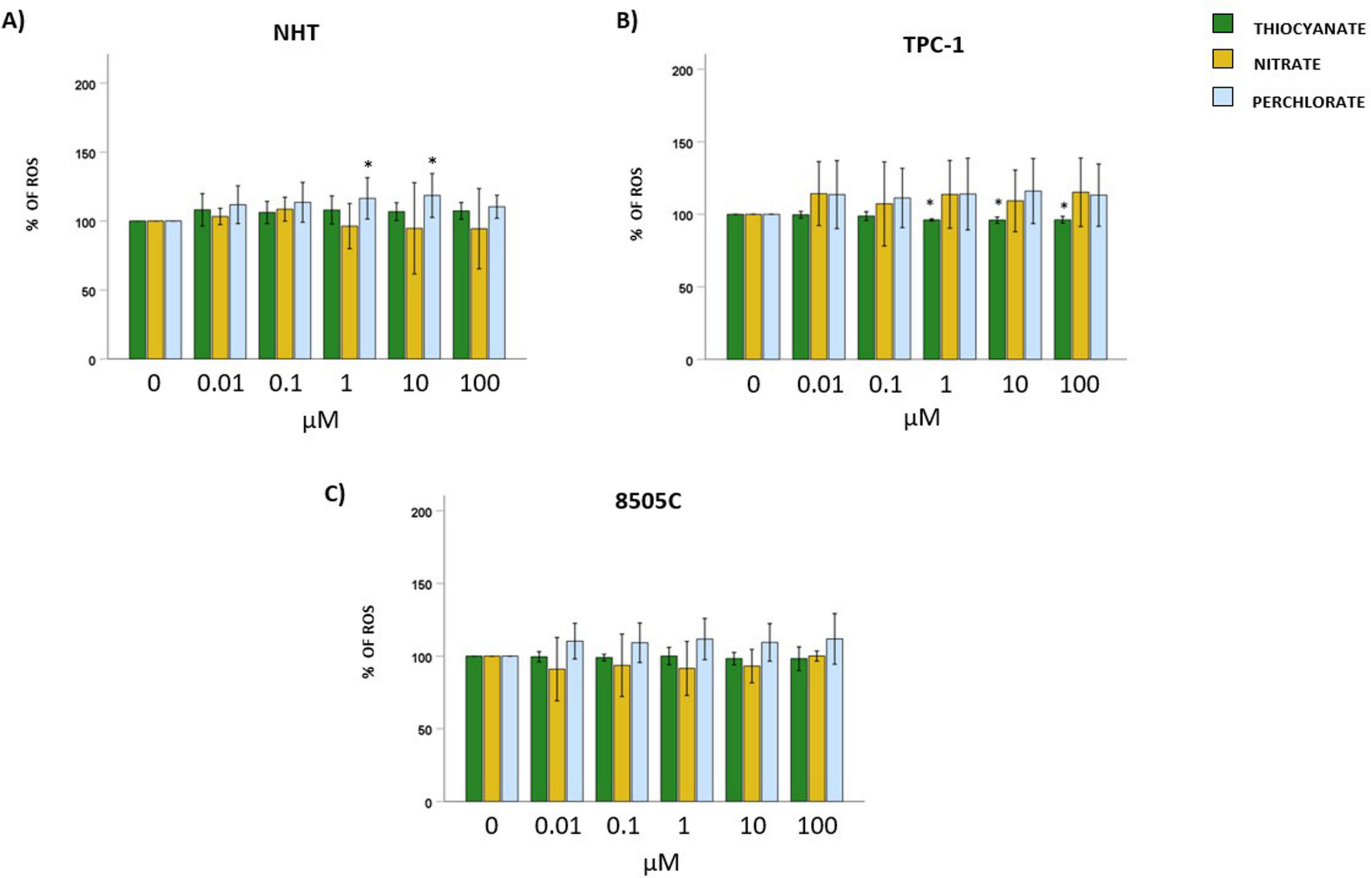
Effect of thiocyanate, nitrate and perchlorate on ROS production. Thyroid cells were treated with increasing concentrations of thiocyanate, nitrate and perchlorate (µM) for 24 hours. Intracellular ROS levels were quantified using a H_2_DCFDA-based fluorescence assay. Results are expressed as percentage of ROS levels in untreated control cells (0 µM), set as 100%. Bar graphs: y-axis indicates the % of total ROS; x-axis indicates treatment with thiocyanate, nitrate or perchlorate (µM). Bars show the effect of thiocyanate (green bars), nitrate (yellow bars) and perchlorate (blue bars) on ROS production. **(A)** shows the effect of thiocyanate, nitrate and perchlorate on total ROS production (in terms of changes of % of control) in NHT. **(B)** shows the effect of thiocyanate, nitrate and perchlorate on total ROS production (in terms of changes of % of control) in TPC-1. **(C)** shows the effect of thiocyanate, nitrate and perchlorate on total ROS production (in terms of changes of % of control) in 8505C. Bars represent the mean ± SD of normalized values. Control (0 µM) values were set to 100% after normalization; as a result, SD is not shown for control bars. Results were expressed as % of cell proliferation relative to control. Significant changes between treated samples *vs.* controls were indicated by *.

Cells were exposed to increasing concentrations of thiocyanate, nitrate, or perchlorate for 24 hours to assess changes in total ROS levels. The induction of ROS in each cell type was confirmed by exposure to H_2_O_2_ (*data not shown*). In NHT, the exposure to thiocyanate or nitrate did not modify ROS levels (ANOVAs: thiocyanate F = 2.063, *p*=NS; nitrate F = 1.133, *p*=NS), while exposure to perchlorate induced a biphasic effect on ROS levels, being increased only by 1 µM (116.5 ± 15% of ROS) and 10 µM (118.5 ± 15.9% of ROS) concentrations (ANOVA: perchlorate F = 3.760, *p* < 0.05; *post hoc* by Bonferroni 1 µM and 10 µM *vs.* 0 µM, *p* < 0.05) (**Figure 3A**). Moving to what was observed in TPC-1, the exposure to thiocyanate reduced ROS levels starting from 1 µM (ANOVA: F = 7.640, *p* < 0.05; *post hoc* by Bonferroni 1, 10, 100 µM *vs.* 0 µM, *p* < 0.05, with the following percentage of ROS 1 µM: 96.1 ± 0.7%; 10 µM 96.0 ± 2.2%; 100 µM: 96.2 ± 2.3%). Exposure to nitrate or perchlorate did not change ROS levels (ANOVAs: nitrate: F = 0.631, *p*=NS; perchlorate F = 0.708, *p*=NS) (**Figure 3B**). Finally, in 8505C, the exposure to all three anions did not induce significant changes in the ROS levels (ANOVAs: thiocyanate F = 0.337, *p* = 0.889; nitrate F = 0.843, *p* = 0.524; perchlorate F = 1.407, *p* = 0.233) (**Figure 3C****).**

### Effect of thiocyanate, nitrate, or perchlorate on CXCL8 and CXCL10 secretion

CXCL8 and CXCL10 concentrations were measured in cell culture supernatants after 24 hours of exposure to increasing concentrations of thiocyanate, nitrate, or perchlorate.

**Figure 4:**
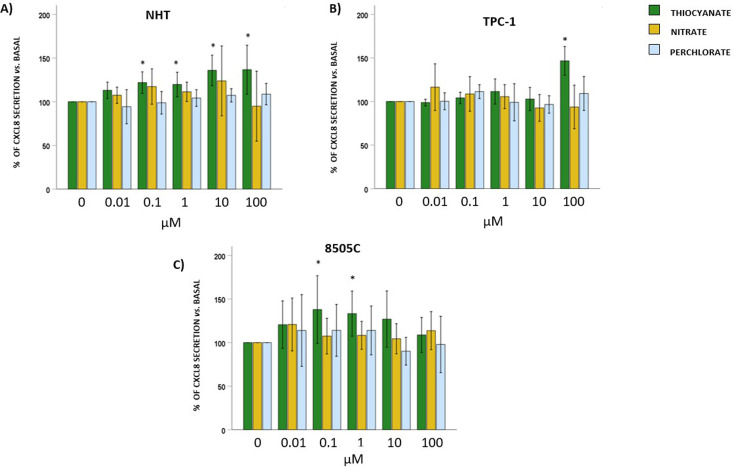
CXCL8 protein levels after exposure of thyroid cells to thiocyanate, nitrate or perchlorate. Results of ELISA assay for the detection of production of CXCL8 chemokine in NHT **(A)**, TPC-1 **(B)** and 8505C **(C)**. Bar graph: y-axis shows the percentage of CXCL8 secretion; x-axis indicates treatment with thiocyanate, nitrate or perchlorate (µM). Bars show the effect of thiocyanate (green bars), nitrate (yellow bars) and perchlorate (blue bars) on CXCL8 secretion. Bars represent the mean ± SD of normalized values. Control (0 µM) values were set to 100% after normalization; as a result, SD is not shown for control bars. Results were expressed as % of cell proliferation relative to control. Significant changes between treated samples *vs.* controls were indicated by *.

Results showed that exposure to thiocyanate increased the basal secretion of CXCL8 by NHT starting from 0.1 µM (ANOVA: thiocyanate F = 10.629, *p* < 0.05; *post hoc* by Bonferroni 0.1, 1, 10 and 100 µM *vs.* 0 µM, *p* < 0.05). No significant changes in CXCL8 secretion were observed after exposure to nitrate (ANOVA: F = 1.883, *p*=NS) or perchlorate (ANOVA: F = 2.186, *p*=NS) (**Figure 4A**).

A similar scenario was observed in TPC-1 cells in which exposure to thiocyanate increased the basal secretion of CXCL8, but only at the highest concentration (ANOVA: thiocyanate F = 22.127, *p* < 0.05; *post hoc* by Bonferroni 100 µM *vs.* 0 µM, *p* < 0.05). No significant changes in CXCL8 secretion were observed after exposure to nitrate (ANOVA: F = 1.870, *p*=NS) or perchlorate (ANOVA: F = 1.549, *p* = 0.197) (**Figure 4B**).

Finally, when 8505C were exposed to each anion, a different pattern of CXCL8 secretion was observed. Indeed, exposure to thiocyanate led to the increase of CXCL8 secretion at the lower concentrations (ANOVA: thiocyanate F = 3.797, *p* < 0.05; *post hoc* by Bonferroni 0.1 and 1 µM *vs.* 0 µM, *p* < 0.05) but not at the higher ones. No significant changes were found when cells were exposed to nitrate (ANOVA: F = 1.111, *p*=NS) or perchlorate (ANOVA: F = 2.181, *p*=NS**) (****Figure 4C**). No detectable concentrations of CXCL10 were observed in all the cell culture supernatants at basal level and no induction of CXCL10 was found after exposure to the three anions (*data not shown*).

### Effect of thiocyanate, nitrate, or perchlorate on CXCL8 and CXCL10 mRNA

Potential changes in mRNA levels of CXCL8 and CXCL10 were evaluated after exposure to thiocyanate, nitrate, or perchlorate for two main reasons: i) to understand if what observed at protein levels was confirmed at the mRNA levels, ii) since that no detectable concentrations were found at protein levels for CXC10, the mRNA of this chemokine was evaluated in order to understand if some changes in CXCL10 could occur.

#### NHT

**Figure 5:**
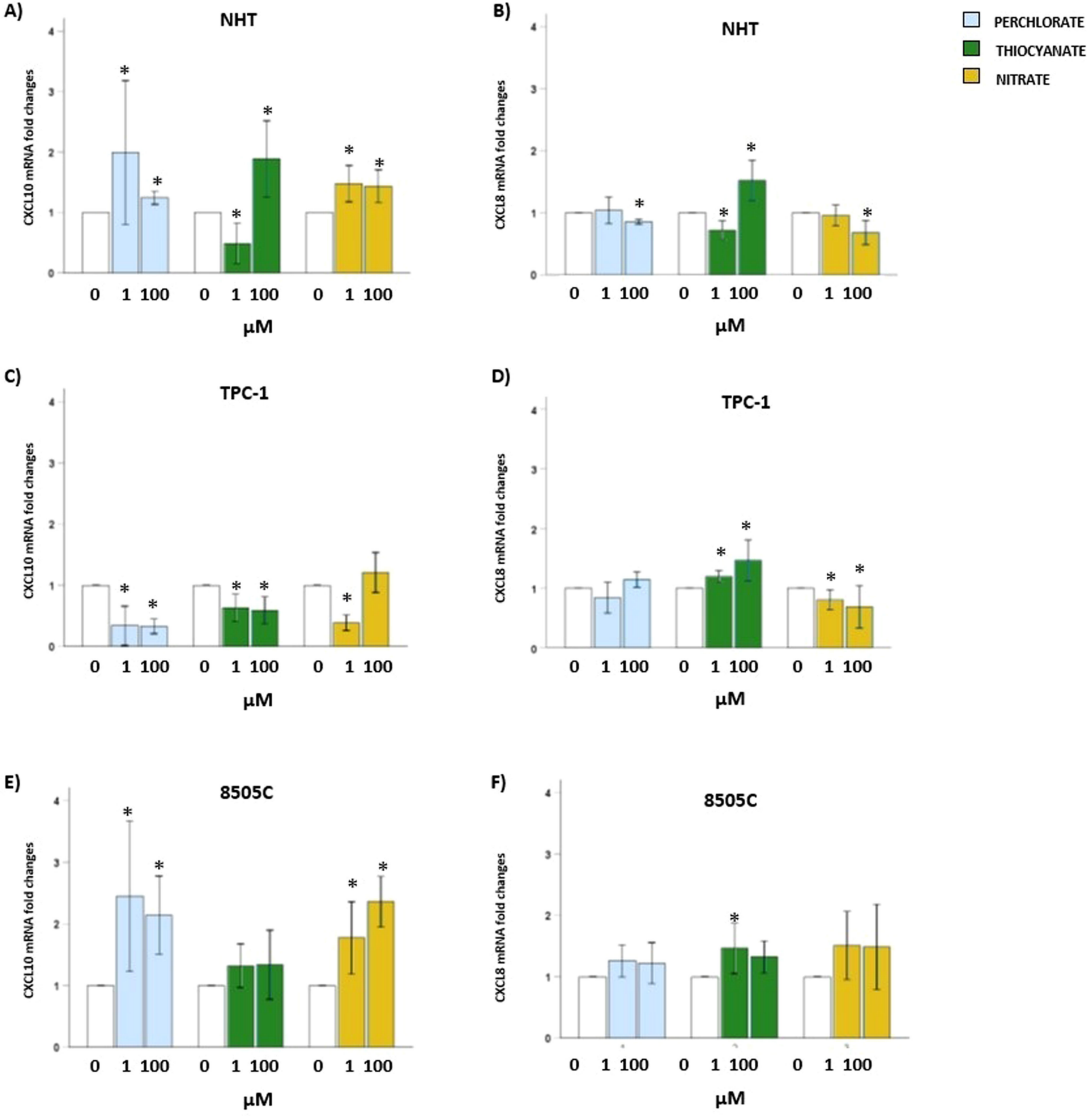
CXCL8 and CXCL10 mRNA levels after exposure of thyroid cells to thiocyanate, nitrate or perchlorate. Results of RT-PCR experiments in NHT **(A, B)**, TPC-1 **(C, D)** and 8505C **(E, F)** cells treated thiocyanate, nitrate or perchlorate (µM). Bar graphs: y-axis indicates the fold changes in the mRNA levels of *CXCL8* and *CXCL10* normalized for *GAPDH* and analysed using the ΔΔCt method (further normalized for untreated samples, see material and methods); x-axis indicate treatments with thiocyanate (green bars), nitrate (yellow bars) or perchlorate (blue bars) at the concentration of 1 and 100 µM. Significant changes between treated samples *vs.* controls were indicated by *.

In NHT (**Figures 5A, B**), exposure to perchlorate 1 and 100 µM led to significant increase in the mRNA of CXCL10 (H = 16.310, *p* < 0.05; *post hoc* by Bonferroni 1 and 100 µM *vs.* 0 µM, *p* < 0.05) and to a significant reduction of CXCL8 mRNA at 100 µM (H = 12.482, *p* < 0.05; *post hoc* by Bonferroni 100 µM *vs.* 0 µM, *p* < 0.05). Exposure to thiocyanate 1 µM led to a significant reduction in the mRNA of CXCL10 whereas thiocyanate 100 µM induced an increase in the mRNA level of CXCL10 (H = 24.023, *p* < 0.05; *post hoc* by Bonferroni 1 and 100 µM *vs.* 0 µM, *p* < 0.05). A similar trend was found when CXCL8 mRNA was analysed, indeed exposure to thiocyanate 1 µM led to significant reduction in the mRNA of CXCL8 whereas thiocyanate 100 µM led to significant increase in the mRNA of CXCL8 (H = 21.365, *p* < 0.05; *post hoc* by Bonferroni 1 and 100 µM *vs.* 0 µM, *p* < 0.05). The exposure to nitrate 1 and 100 µM led to a significant increase in the mRNA of CXCL10 (H = 17.115, *p* < 0.05; *post hoc* by Bonferroni 1 and 100 µM *vs.* 0 µM, *p* < 0.05). Moving to CXCL8, a significant reduction in the mRNA was observed after exposure to nitrate 100 µM (H = 9.875, *p* < 0.05; *post hoc* by Bonferroni 100 µM *vs.* 0 µM, *p* < 0.05).

#### Thyroid cancer cells

In TPC-1 (**Figures 5C, D**), exposure to perchlorate 1 and 100 µM led to significant reduction in the mRNA of CXCL10 (H = 15.981, *p* < 0.05; *post hoc* by Bonferroni 1 and 100 µM *vs.* 0 µM, *p* < 0.05) a not significant change of CXCL8 mRNA was found for 1 and 100 µM *vs.* basal levels (H = 11.620, *p* < 0.05; *post hoc* by Bonferroni 1 and 100 µM *vs.* 0 µM, *p*=NS). Exposure to thiocyanate led to significant reduction in the mRNA of CXCL10 (H = 15.981, *p* < 0.05; *post hoc* by Bonferroni 1 and 100 µM *vs.* 0 µM, *p* < 0.05). An opposite trend was found when CXCL8 mRNA was analysed, indeed exposure to thiocyanate 1 and 100 µM led to significant increase in the mRNA of CXCL8 (H = 16.573, *p* < 0.05; *post hoc* by Bonferroni 1 and 100 µM *vs.* 0 µM, *p* < 0.05). The exposure to nitrate 1 µM led to significant reduction in the mRNA of CXCL10 (H = 15.845, *p* < 0.05; *post hoc* by Bonferroni 1 µM *vs.* 0 µM, *p* < 0.05). Moving to CXCL8 a significant reduction in the mRNA was observed after exposure to nitrate (H = 10.907, *p* < 0.05; *post hoc* by Bonferroni 1 and 100 µM *vs.* 0 µM, *p* < 0.05).

Finally, in 8505C (**Figures 5E, F**) exposure to perchlorate 1 and 100 µM led to significant increase in the mRNA of CXCL10 (H = 18.128, *p* < 0.05; *post hoc* by Bonferroni 1 and 100 µM *vs.* 0 µM, *p* < 0.05). A not significant trend toward an increase of CXCL8 mRNA was found for 1 and 100 µM *vs.* basal levels (H = 6.607, *p* < 0.05; *post hoc* by Bonferroni 1 and 100 µM *vs.* 0 µM, *p*=NS). Exposure to thiocyanate did not modify the mRNA of CXCL10 (H = 2.485, *p*=NS). When CXCL8 mRNA was analysed, exposure to thiocyanate 1 µM led to significant increase in the mRNA (H = 11.770, *p* < 0.05; *post hoc* by Bonferroni 1 µM *vs.* 0 µM, *p* < 0.05). The exposure to nitrate led to a significant increase in the mRNA of CXCL10 (H = 15.325, *p* < 0.05; *post hoc* by Bonferroni 1 and 100 µM *vs.* 0 µM, *p* < 0.05). Moving to CXCL8 a not significant modification in the mRNA was observed after exposure to nitrate (H = 3.793, *p*=NS).

### Effect of thiocyanate on thyroid cells migration

**Figure 6:**
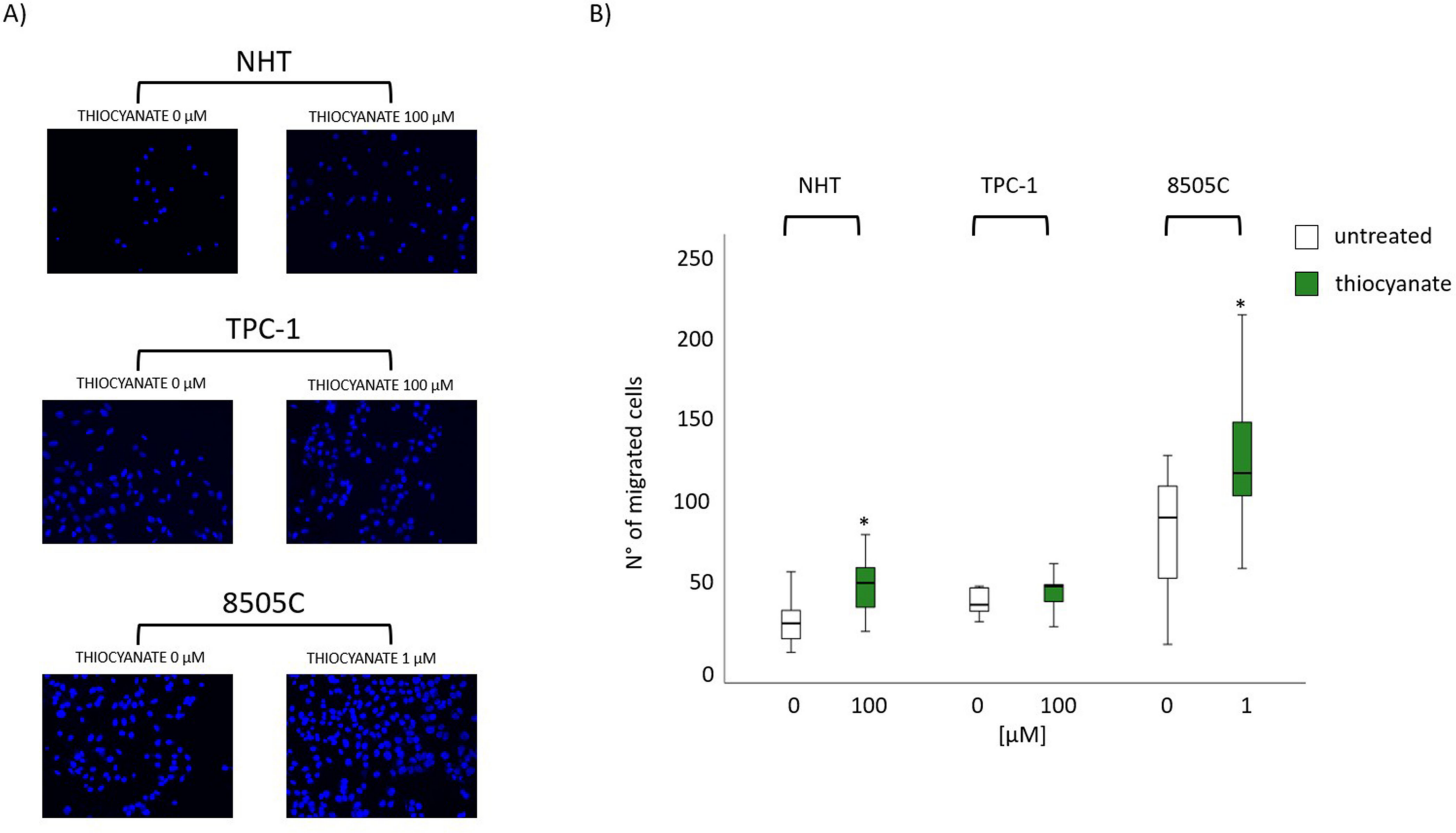
NHT, TPC-1 and 8505C migration after treatment with thiocyanate. Results of migration assay with transwell-migration chamber system. **(A)** Representative images and after 16 hours of migration within the trans-well migration chamber system for NHT (thiocyanate 0 µM and 100 µM), TPC-1 (thiocyanate 0 µM and 100 µM) and 8505C (thiocyanate 0 µM and 1 µM), cell nuclei were stained with Hoecst 33342. **(B)** Box plot of the mean number of migrated cells basally (white boxes) and after treatment with thiocyanate (green boxes) 0 µM and 1 µM (for 8505C) and 100 µM (for NHT and TPC-1). * indicates significant values (*p* < 0.05 Wilcoxon test). The centre line indicates the median value (50th percentile), while the box contains the 25th to 75th percentiles of data. The black whiskers mark the 5th and 95th percentiles.

Cell migration assays were performed in NHT, TPC-1 and 8505C after 24h treatment with thiocyanate. As shown in **Figures 6A, B** thiocyanate treatment at the same concentration at which it induced CXCL8 secretion was observed in NHT and 8505C but not in TPC-1.

## Discussion

This study is the first to evaluate whether perchlorate, nitrate and thiocyanate can directly induce changes in chemokine expression or oxidative stress in cultured human thyroid cells. These findings represent a first step in redefining the biological impact of these common environmental chemicals on thyroid cells, beyond interfering with iodide uptake.

Exposure to thiocyanate, nitrate, or perchlorate did not affect cell viability in normal human thyrocytes or the anaplastic thyroid cancer line 8505C. However, TPC-1 cells showed reduced viability after high-dose thiocyanate exposure, with no changes by either nitrate or perchlorate. These results suggest minimal cytotoxicity only for thiocyanate, with TPC-1 cells being more susceptible. Exposure to thiocyanate and perchlorate had no effect on NHT cell proliferation, while high-dose of nitrate induced a slight but significant induction of proliferation. In TPC-1 only high-dose of perchlorate increased proliferation; thiocyanate and nitrate had no significant effects. In 8505C cells, only high concentrations of thiocyanate enhanced proliferation, while nitrate and perchlorate showed no impact. Overall, the three anions exhibit minimal pro-proliferative properties, with a certain effect registered only for high levels of exposure. Each anion elicited a distinct pattern of response, and these patterns were not uniform across normal thyrocytes, differentiated thyroid cancer cells, and anaplastic thyroid cancer cells. Based on these results, the effects of perchlorate, nitrate, and thiocyanate on cell viability and proliferation seem to be dependent on both the specific compound and the cellular model being examined.

Oxidative stress assays indicated that perchlorate increased ROS at intermediate doses in NHT but not at low or high concentrations. On the other hand, no effect was found on ROS production when NHT were exposed to thiocyanate or nitrate. In TPC-1 cells, thiocyanate reduced ROS at medium-to-high concentrations, while nitrate and perchlorate did not affect ROS production. In 8505C cells, none of the compounds significantly altered ROS levels.

Moving to the investigation of chemokine secretion, thiocyanate was the only anion that consistently increased CXCL8 secretion in both NHT and TPC-1 cells, though at different concentrations. In 8505C cells, thiocyanate only at lower concentrations increased CXCL8 levels, with no effect at higher concentrations. Nitrate and perchlorate did not affect CXCL8 secretion in any type of thyroid cells. CXCL10 was undetectable at baseline and not induced by any anions. The design of the present study does not allow to draw mechanistic conclusions. The observed divergence between ROS patterns and CXCL8 secretion, particularly after thiocyanate exposure, may reflect the heterogeneous genetic background of the cell models. These results could suggest that ROS modulation and CXCL8 production in thyroid cells, should be regarded, at least in part, as uncoupled processes. In addition, as reported by the literature, ROS can exert bidirectional effects on inflammatory signalling depending on intracellular redox balance (32). However, it should be highlighted that only specifically designed mechanistic studies will fully elucidate this issue.

Given the undetectable protein levels of CXCL10, mRNA of both CXCL10 and CXCL8 was analysed after exposure to the three anions. Distinct patterns emerged across various cell lines and specific anions at different concentrations. Thiocyanate increased CXCL8 mRNA in all cell types, consistent with protein data, except for a reduction at 1 µM in NHT. For CXCL10, thiocyanate had no effect in 8505C cells, induced a biphasic response in NHT (reduction at 1 µM and increase at 100 µM), and caused an overall downregulation in TPC-1 cells. Nitrate reduced CXCL8 mRNA in NHT and TPC-1 without affecting protein secretion, while increasing CXCL10 mRNA in NHT and 8505C and reducing it in TPC-1. Perchlorate reduced CXCL8 mRNA in NHT (not observed at the protein level) and contributed to CXCL10 upregulation in NHT and 8505C, while decreasing it in TPC-1.

The observed discrepancies between mRNA expression and secreted protein levels are likely due to post-transcriptional regulation. Indeed, chemokine production is regulated at multiple levels, including mRNA stability, translational efficiency, and secretion dynamics. For instance, CXCL8 can be stored in intracellular compartments such as Golgi-associated vesicles and Weibel-Palade bodies, which are subject to regulated exocytosis (33, 34). Consequently, mRNA levels do not necessarily correlate with extracellular protein abundance, as previously reported for chemokines (35). These findings suggest that the three anions may differentially affect these regulatory steps, although dedicated mechanistic studies are needed to confirm this hypothesis. In both cases, the EDCs typical “non-monotonic” modulation of gene expression is evident. Indeed, the impact of varying concentrations of anions is not always uniform. The down-regulation of a specific gene at low concentrations, followed by up-regulation or no change at higher concentrations, indicates a non-monotonic, biphasic, or U-shaped dose-response curve typical of EDCs.

This chemokine profile induced by inorganic anions supports the concept of a functional modulation of the thyroid tumour microenvironment. Indeed, at the protein level, a preferential upregulation of CXCL8 is observed, with thiocyanate emerging as the most consistent inducer of its secretion. CXCL8 is a key mediator involved in angiogenesis, extracellular matrix remodelling, and tumour progression, and its selective increase suggests a shift toward a more pro-tumorigenic inflammatory milieu. In contrast, CXCL10 remains undetectable at the protein level under all experimental conditions. However, mRNA analyses reveal that CXCL10 transcription is increased in specific experimental settings, suggesting the activation of an immunosurveillance-related transcriptional program.

In order to assess if thiocyanate, likely through increased CXCL8 secretion, also promotes cell migration, NHT, TPC-1, and 8505C cell lines were treated with thiocyanate at a concentration that increased CXCL8 levels. The results of migration assays showed that an increase in cell migration was observed following treatment with thiocyanate in NHT and 8505C cell lines but not in TPC-1. This would fit with the notion that CXCL8 although crucial is not the only mediator of cell migration which is indeed a multifactorial event (36). These findings would suggest that thiocyanate could promote pro-tumorigenic effect in relation with a specific cell type.

Collectively, these findings point to a scenario in which inorganic anions actively reshape the TME by reinforcing pro-tumorigenic chemokine signalling while only weakly or incompletely engaging anti-tumour immune pathways. To our knowledge, this is the first study demonstrating that perchlorate and thiocyanate, but not nitrate, can directly modulate ROS production and chemokine secretion in human thyroid cells (both normal and malignant). Previous research has largely framed these anions exclusively as NIS inhibitors, with expected consequences for iodide uptake and thyroid hormone synthesis. However, our data show that their actions extend beyond this classical mechanism.

Epidemiological studies show inconsistent results regarding the impact of these anions on thyroid function in the general population. While several cohorts report associations between urinary concentrations of perchlorate, nitrate and thiocyanate and altered TSH or FT4 levels, others fail to confirm significant effects, especially at environmentally relevant exposures. Parallel to this, an increasing number of studies have raised concerns about a potential link between chronic nitrate exposure and the risk of thyroid cancer, although available data remain heterogeneous. This highlight that the biological actions of NIS-inhibiting anions may not be fully captured by their classical “goitrogenic” role. Notably, recent evidence indicates that these anions can exert biological effects unrelated to NIS inhibition, supporting the hypothesis that they may activate additional molecular targets (1). Dedicated mechanistic studies (such as NIS-silencing experiments, uptake quantification, or pathway-specific inhibition) will be essential to clarify whether intracellular accumulation via NIS is necessary for triggering redox and inflammatory responses, or whether alternative transporters and signalling cascades are involved. In conclusions, the present findings introduce a previously unrecognized layer of complexity in the toxicological profile of these widespread environmental contaminants. Our data suggest that, among the three anions tested, perchlorate and in particularly thiocyanate promote redox remodelling and a CXCL8-driven inflammatory state.

## Data Availability

The raw data supporting the conclusions of this article will be made available by the authors, without undue reservation.

## References

[1] Serrano-Nascimento C Nunes MT . Perchlorate, nitrate, and thiocyanate: environmental relevant NIS-inhibitors pollutants and their impact on thyroid function and human health. Front Endocrinol (Lausanne). (2022) 13:995503. doi: 10.3389/fendo.2022.995503. PMID: 36339434 PMC9633673

[2] Caiano A Ramos AS Vieira MT . NiTi wires coated by nanomultilayers - a solution for self-healing? Microsc Microanal. (2015) 21:11–2. doi: 10.1017/s1431927615013860. PMID: 26227686

[3] Ma L Hu L Feng X Wang S . Nitrate and nitrite in health and disease. Aging Dis. (2018) 9:938–45. doi: 10.14336/ad.2017.1207. PMID: 30271668 PMC6147587

[4] King L Wang Q Xia L Wang P Jiang G Li W . Environmental exposure to perchlorate, nitrate and thiocyanate, and thyroid function in Chinese adults: a community-based cross-sectional study. Environ Int. (2023) 171:107713. doi: 10.1016/j.envint.2022.107713. PMID: 36565572

[5] Calderón R Jara C Albornoz F Palma P Arancibia-Miranda N Karthikraj R . Exploring the destiny and distribution of thiocyanate in the water-soil-plant system and the potential impacts on human health. Sci Total Environ. (2022) 835:155502. doi: 10.1016/j.scitotenv.2022.155502 35490807

[6] Braverman LE He X Pino S Cross M Magnani B Lamm SH . The effect of perchlorate, thiocyanate, and nitrate on thyroid function in workers exposed to perchlorate long-term. J Clin Endocrinol Metab. (2005) 90:700–6. doi: 10.1210/jc.2004-1821. PMID: 15572417

[7] Lisco G Accardo G Pupilli C Malandrino P De Geronimo V Triggiani V . Perchlorates in the treatment of hyperthyroidism and thyrotoxicosis: a comprehensive review. Endocrine. (2024) 85:1–10. doi: 10.1007/s12020-023-03679-y. PMID: 38195966 PMC11246303

[8] Morgan PE Pattison DI Talib J Summers FA Harmer JA Celermajer DS . High plasma thiocyanate levels in smokers are a key determinant of thiol oxidation induced by myeloperoxidase. Free Radic Biol Med. (2011) 51:1815–22. doi: 10.1016/j.freeradbiomed.2011.08.008. PMID: 21884783

[9] Willemin ME Lumen A . Thiocyanate: a review and evaluation of the kinetics and the modes of action for thyroid hormone perturbations. Crit Rev Toxicol. (2017) 47:537–63. doi: 10.1080/10408444.2017.1281590. PMID: 28632039

[10] Isaac E Pfeffer PL . Growing cattle embryos beyond day 8 - an investigation of media components. Theriogenology. (2021) 161:273–84. doi: 10.1016/j.theriogenology.2020.12.010. PMID: 33360161

[11] Soto AM Sonnenschein C . Environmental causes of cancer: endocrine disruptors as carcinogens. Nat Rev Endocrinol. (2010) 6:363–70. doi: 10.1038/nrendo.2010.87. PMID: 20498677 PMC3933258

[12] Ward MH Kilfoy BA Weyer PJ Anderson KE Folsom AR Cerhan JR . Nitrate intake and the risk of thyroid cancer and thyroid disease. Epidemiology. (2010) 21:389–95. doi: 10.1097/ede.0b013e3181d6201d. PMID: 20335813 PMC2879161

[13] Zhang L Fang C Liu L Liu X Fan S Li J . A case-control study of urinary levels of iodine, perchlorate and thiocyanate and risk of papillary thyroid cancer. Environ Int. (2018) 120:388–93. doi: 10.1016/j.envint.2018.08.024. PMID: 30125856

[14] Wang H Jiang Y Song J Liang H Liu Y Huang J . The risk of perchlorate and iodine on the incidence of thyroid tumors and nodular goiter: a case-control study in southeastern China. Environ Health. (2022) 21:4. doi: 10.1186/s12940-021-00818-8. PMID: 34980104 PMC8725411

[15] Shiue I . Urinary thiocyanate concentrations are associated with adult cancer and lung problems: US NHANES, 2009-2012. Environ Sci pollut Res Int. (2015) 22:5952–60. doi: 10.1007/s11356-014-3777-8. PMID: 25367645

[16] Rotondi M Coperchini F Latrofa F Chiovato L . Role of chemokines in thyroid cancer microenvironment: is CXCL8 the main player? Front Endocrinol (Lausanne). (2018) 9:314. doi: 10.3389/fendo.2018.00314. PMID: 29977225 PMC6021500

[17] Ameziane El Hassani R Buffet C Leboulleux S Dupuy C . Oxidative stress in thyroid carcinomas: biological and clinical significance. Endocr Relat Cancer. (2019) 26:R131–43. doi: 10.1530/erc-18-0476. PMID: 30615595

[18] Coperchini F De Marco G Croce L Denegri M Greco A Magri F . PFOA, PFHxA and C6O4 differently modulate the expression of CXCL8 in normal thyroid cells and in thyroid cancer cell lines. Environ Sci pollut Res Int. (2023) 30:63522–34. doi: 10.1007/s11356-023-26797-6. PMID: 37052835

[19] Rotondi M Coperchini F Pignatti P Sideri R Groppelli G Leporati P . Interferon-γ and tumor necrosis factor-α sustain secretion of specific CXC chemokines in human thyrocytes: a first step toward a differentiation between autoimmune and tumor-related inflammation? J Clin Endocrinol Metab. (2013) 98:308–13. doi: 10.1210/jc.2012-2555. PMID: 23118425

[20] Coperchini F Greco A Teliti M Denegri M Croce L Calì B . *In vitro* study of the UV-filter homosalate effects on rat and human thyroid cells. Environ pollut. (2024) 363:125063. doi: 10.1016/j.envpol.2024.125063. PMID: 39366447

[21] Coperchini F Greco A Croce L Teliti M Calì B Chytiris S . Do PFCAs drive the establishment of thyroid cancer microenvironment? Effects of C6O4, PFOA and PFHxA exposure in two models of human thyroid cells in primary culture. Environ Int. (2024) 187:108717. doi: 10.1016/j.envint.2024.108717. PMID: 38728818

[22] Coperchini F Pignatti P Carbone A Bongianino R Di Buduo CA Leporati P . TNF-α increases the membrane expression of the chemokine receptor CCR6 in thyroid tumor cells, but not in normal thyrocytes: potential role in the metastatic spread of thyroid cancer. Tumour Biol. (2016) 37:5569–75. doi: 10.1007/s13277-015-4418-7. PMID: 26577851

[23] Snyder SA Vanderford BJ Rexing DJ . Trace analysis of bromate, chlorate, iodate, and perchlorate in natural and bottled waters. Environ Sci Technol. (2005) 39:4586–93. doi: 10.1021/es047935q. PMID: 16047796

[24] Srinivasan A Viraraghavan T . Perchlorate: health effects and technologies for its removal from water resources. Int J Environ Res Public Health. (2009) 6:1418–42. doi: 10.3390/ijerph6041418. PMID: 19440526 PMC2681191

[25] Leung AM Pearce EN Braverman LE . Perchlorate, iodine and the thyroid. Best Pract Res Clin Endocrinol Metab. (2010) 24:133–41. doi: 10.1016/j.beem.2009.08.009. PMID: 20172477 PMC4137763

[26] Qin X Zhang T Gan Z Sun H . Spatial distribution of perchlorate, iodide and thiocyanate in the aquatic environment of Tianjin, China: environmental source analysis. Chemosphere. (2014) 111:201–8. doi: 10.1016/j.chemosphere.2014.03.082. PMID: 24997919

[27] Eminedoki DG Monanu MO Anosike EO . Thiocyanate levels of mainly dietary origin in serum and urine from a human population sample in Port Harcourt, Nigeria. Plant Foods Hum Nutr. (1994) 46:277–85. doi: 10.1007/bf01088426. PMID: 7716108

[28] Kirk AB Dyke JV Martin CF Dasgupta PK . Temporal patterns in perchlorate, thiocyanate, and iodide excretion in human milk. Environ Health Perspect. (2007) 115:182–6. doi: 10.1289/ehp.9558. PMID: 17384762 PMC1817678

[29] European Environment Agency (EEA) . . 10.1007/BF02986943 24234300

[30] EEA . (2025). European Environment Agency (EEA.

[31] Crowley LC Christensen ME Waterhouse NJ . Measuring survival of adherent cells with the colony-forming assay. Cold Spring Harb Protoc. (2016) 2016:721–4. doi: 10.1101/pdb.prot087171. PMID: 27480717

[32] Nakajima S Kitamura M . Bidirectional regulation of NF-κB by reactive oxygen species: a role of unfolded protein response. Free Radic Biol Med. (2013) 65:162–74. doi: 10.1016/j.freeradbiomed.2013.06.020. PMID: 23792277

[33] Rotondi M Coperchini F Chiovato L . CXCL8 in thyroid disease: from basic notions to potential applications in clinical practice. Cytokine Growth Factor Rev. (2013) 24:539–46. doi: 10.1016/j.cytogfr.2013.08.001. PMID: 24011840

[34] Hol J Küchler AM Johansen FE Dalhus B Haraldsen G Oynebråten I . Molecular requirements for sorting of the chemokine interleukin-8/CXCL8 to endothelial Weibel-Palade bodies. J Biol Chem. (2009) 284:23532–9. doi: 10.1074/jbc.m900874200. PMID: 19578117 PMC2749127

[35] Fan J Heller NM Gorospe M Atasoy U Stellato C . The role of post-transcriptional regulation in chemokine gene expression in inflammation and allergy. Eur Respir J. (2005) 26:933–47. doi: 10.1183/09031936.05.00120204. PMID: 16264057

[36] de Visser KE Joyce JA . The evolving tumor microenvironment: from cancer initiation to metastatic outgrowth. Cancer Cell. (2023) 41:374–403. doi: 10.1016/j.ccell.2023.02.016. PMID: 36917948

